# Supplementary material for: What Levers to Promote Teachers’ Wellbeing during the COVID-19 Pandemic and Beyond: Lessons Learned from a 2021 Online Study in Six Countries
Source: Int J Environ Res Public Health. 2022 Jul 27;19(15):9151. doi: 10.3390/ijerph19159151 (PMC9368544; doi:10.3390/ijerph19159151)
Supplement: Supplementary file 1 [file ijerph-19-09151-s001.zip › ijerph-1804042-supplementary.pdf]

Table S1. Pearson correlations* between five dimensions of teachers' wellbeing in France (n= 3,646), Québec (n=2,347) and Belgium (n=1,268), 2021 International Barometer of Education Personnel's Health and Wellbeing (I-BEST)

	Job satisfaction	Work/life balance	Subjective health	Mental health	Life satisfaction
**Job satisfaction**	1.00				
**Work/life balance**	0.50	1.00			
**Subjective health**	0.36	0.35	1.00		
**Mental health**	0.43	0.55	0.38	1.00	
**Life satisfaction**	0.50	0.46	0.47	0.50	1.00

* Weighted statistics taking into account gender and level of education + age group in France and Belgium

Table S2. List of the 29 potential determinants of teacher's wellbeing that were considered in the forward stepwise regression procedure in addition of age and gender, 2021 International Barometer of Education Personnel's Health and Wellbeing (I-BEST)

	Name variable	Question	Response items	Format in the analysis and reference group
**Private life**				
	Household partner	In your household, you live with partner?	Yes, no	2 categories: yes, no (ref.)
	Household child(ren)	In your household, you live with children?	Yes, no	2 categories: yes, no (ref.)
	Access to healthcare	Are you satisfied with your access to healthcare in your country?	Very satisfied, satisfied, neither satisfied nor dissatisfied, dissatisfied, very dissatisfied	3 categories: very satisfied/satisfied (ref.), neither satisfied nor dissatisfied, dissatisfied/very dissatisfied
	Access to training/prevention resources	Do you have access to training and/or prevention resources on health and well-being?	Yes, no	2 categories: yes (ref.), no
**Professional life**				
General factors	Age of students	In your main school, what age group do your students belong to?	3-5 years old, 6-11 years old, 11-15 years old, 16-18 years old	4 categories: 3-5, 6-11, 11-15, 16-18 years old (ref.)
	Seniority	How many years have you worked in the education sector? (In total, including the current year)	1 year, 2 to 4 years, 5 to 9 years, 10 to 14 years, 15 to 19 years, 20 to 30 years, over 30 years	3 categories: <5 (ref.), 5-30, >30 years
	Employment status	Do you work… ?	Full-time, part-time	2 categories: full-time (ref.), part-time
	Union membership	Do you have a membership in a union?	Yes, no, do not want to answer	3 categories: yes (ref.), no, do not want to answer
	Remote work	Do you currently teach remotely?	Yes, totally; yes, partially; no	2 categories: at least partially, no (ref.)
	School sector	Your school is…	Public, private	2 categories: public (ref.), private
Physical environment	School size	How many students are in your school?	Less than 100 students, between 100 and 199 students, between 200 and 799 students, more than 800 students	4 categories: <100 (ref.), 100-199, 200-799, >800
	School urbanicity	Your school is located in an environment that is rather?	6-point Likert-scale from "A: very urban" to "F: very rural"	3 categories: urban (A-B) (ref.), intermediate (C-D), rural (E-F)
	School facilities	Are you satisfied with the school facilities	6-point Likert-scale: from "A: very satisfied" to "F: very dissatisfied"	2 categories: satisfied (A-C) (ref.), unsatisfied (D-F)
	Material conditions	Are your satisfied with the material conditions (workspaces, teaching materials, Internet)?	6-point Likert-scale: from "A: very satisfied" to "F: very dissatisfied"	2 categories: satisfied (A-C) (ref.), unsatisfied (D-F)
	Indoor air quality	Are your satisfied with the air quality inside the buildings?	6-point Likert-scale: from "A: very satisfied" to "F: very dissatisfied"	2 categories: satisfied (A-C) (ref.), unsatisfied (D-F)
	Indoor noise level	Are your satisfied with the sound level inside the buildings?	6-point Likert-scale: from "A: very satisfied" to "F: very dissatisfied"	2 categories: satisfied (A-C) (ref.), unsatisfied (D-F)

	Name variable	Question	Response items	Format in the analysis and reference group
Psychosocial environment	Feeling of safety at school	Do you feel safe at your workplace?	Always, most of the time, rarely, not at all	3 categories: always (ref.), most of the time, rarely/not at all
	Victim of violence	Have you been the victim of workplace violence in the past 12 months?	Yes, no	2 categories: yes, no (ref.)
	Witness of violence	At work, have you witnessed violence in the past 12 months?	Yes, no	2 categories: yes, no (ref.)
	Quality of relationships with students	In your current work, how do you rate the quality of your relationship with pupils	6-point Likert scale from "A: very bad" to "F: very good"	3 categories: low, medium, high (ref.)[1]
		In your current work, how do you rate the level of disciplinary of pupils	6-point Likert scale from "A: very bad" to "F: very good"	
	Quality of relationships with colleagues	If needed, in your school, do you get support from your colleagues?	Yes, no	3 categories: low, medium, strong (ref.)[2]
		In your current work, how do you rate the quality of your relationship with your colleagues	6-point Likert scale from "A: very bad" to "F: very good"	
	Quality of relationships with superiors	If needed, in your school, do you get support from your superiors?	Yes, no	3 categories: low, medium, strong (ref.)[3]
		To what extent are the supervisory authorities concerned with the health and well-being of the staff?	Not at all concerned, a little concerned, fairly concerned, very concerned	
		Is your work recognized and valued by the school administration and/or superiors?	Yes, always; from time to time; rarely; No, never	
Organization / career perspectives	Autonomy at work	How much autonomy do you have in your work?	A lot, some, little, zero	3 categories: a lot (ref.), some, little/zero
	Teamwork	In your school, do you make important decisions as a team?	6-point Likert scale from "A: always" to "F: never"	2 categories: always-often(A-C) (ref.), rarely-never (D-F)
	Communication	At work, are you informed sufficiently in advance about, for example, important decisions, changes, or future projects?	6-point Likert scale from "A: always" to "F: never"	2 categories: always-often(A-C) (ref.), rarely-never (D-F)
	Societal appreciation	I have the impression that being a teacher is valued by society	Strongly disagree, disagree, agree, strongly agree	2 categories: disagree/strongly disagree, agree/strongly agree (ref.)
	Salary	In your current work, how do you rate your salary?	6-point Likert scale from "A: very good" to "F: very bad"	2 categories: satisfied (A-C) (ref.), unsatisfied (D-F)
	Continuing education	In your current work, how do you rate the possibilities of training?	6-point Likert scale from "A: very good" to "F: very bad"	2 categories: satisfied (A-C) (ref.), unsatisfied (D-F)
	Career progression	Evaluation of the possibilities of promotion	6-point Likert scale from "A: very good" to "F: very bad"	2 categories: satisfied (A-C) (ref.), unsatisfied (D-F)

[1] A composite score based on the sum of the two 6-point Likert scales and then divided in tertiles; [2] a composite score based on the combination of the two questions and then divided in tertiles. [3] a composite score based on the combination of the three questions and then divided in tertiles.

Table S3. Factors* associated with job satisfaction among French, Québec and Belgian teachers, 2021 International Barometer of Education Personnel's Health and Wellbeing (I-BEST)

		France		Québec		Belgium	
% Job satisfaction#		*13.4%*		*25.3%*		*27.6%*	
		**β**	**p-value**	**β**	**p-value**	**β**	**p-value**
**Sociodemographic**							
Gender	*Men*	-0.49	0.001				
	Women	Ref.					
**Private life**							
Access to healthcare	*Dissatisfied/very dissatisfied*	-0.74	0.001	-0.37			
	Neither satisfied nor dissatisfied	-0.49	0.003	-0.40			
	Very satisfied/satisfied	Ref.		Ref.			
**Professional life**							
General factors							
Age of students	*3-5*			-0.35	0.093	-0.91	<0.001
	6-11			-0.55	<0.001	-0.59	0.001
	11-15			-0.24	0.109	-0.31	0.091
	16-18 years old			Ref.		Ref.	
Seniority	*<5*	Ref.					
	5-30	-0.74	<0.001				
	> 30 years	-0.99	<0.001				
Employment status	*Full-time*					Ref.	
	Part-time					-0.58	0.011
Union membership	*Yes*			Ref.			
	No			-1.17	0.006		
	Do not want to answer			0.58	0.338		
Physical environment							
Material conditions	*Unsatisfied (D-F)*	-0.33	0.003				
	Satisfied (A-C)	Ref.					
Indoor air quality	*Unsatisfied (D-F)*			0.34	0.002		
	Satisfied (A-C)			Ref.			
Psychosocial environment							
Feeling of safety at school	*Rarely/not at all*	-1.49	<0.001			-0.74	0.009
	Most of the time	-0.39	<0.001			-0.39	0.011
	Always	Ref.				Ref.	
Victim of violence	*Yes*	-0.30	0.025				
	No	Ref.					
Quality of relationships with students	*Low*	-1.05	<0.001	-0.93	<0.001	-0.93	<0.001
	Medium	-0.49	<0.001	-0.23	0.038	-0.59	0.001
	Strong	Ref.		Ref.		Ref.	
Quality of relationships with superiors	*Low*	-0.70	<0.001	-0.50	0.002	-0.63	<0.001
	Medium	-0.37	0.006	-0.30	0.025	-0.39	0.028
	Strong	Ref.		Ref.		Ref.	
**Organization / career perspectives**							
Autonomy at work	*Little/zero*	-0.59	0.032	-0.99	<0.001	-1.08	<0.001
	Some	-0.49	<0.001	-0.40	<0.001	-0.67	<0.001
	A lot	Ref.		Ref.		Ref.	
Societal appreciation	*Disagree/strongly disagree*	-0.74	0.004	-1.03	<0.001		

% Job satisfaction#		France<br>*13.4%*		Québec<br>*25.3%*		Belgium<br>*27.6%*	
		**β**	**p-value**	**β**	**p-value**	**β**	**p-value**
	Agree/strongly agree	Ref.		Ref.			
Salary	*Unsatisfied (D-F)*	-0.81	<0.001	-0.40	<0.001	-0.87	<0.001
	Satisfied (A-C)	Ref.		Ref.		Ref.	
Continuing education	*Unsatisfied (D-F)*			-0.33	0.008		
	Satisfied (A-C)			Ref.			
Career progression	*Unsatisfied (D-F)*	-0.77	<0.001			-0.48	0.002
	Satisfied (A-C)	Ref.				Ref.	

Weighted statistics taking into account gender and level of education + age group in France and Belgium

*Covariates that were not selected by the forward stepwise logistic regression procedure in any of the 3 country models of job satisfaction: age, household partner, household child(ren), access to training/prevention resources, remote work, school sector / school size, school urbanicity, school facilities, indoor noise level / witness of violence, quality of relationships with colleagues /teamwork, communication

Table S4. Factors* associated with poor work/life balance among French, Québec and Belgian teachers, 2021 International Barometer of Education Personnel's Health and Wellbeing (I-BEST)

		France		Québec		Belgium	
% Poor work/life balance#		*14.6%*		*8.0%*		*9.2%*	
		**β**	**p-value**	**β**	**p-value**	**β**	**p-value**
**Sociodemographic**							
**Age**		-0.01	0.045				
Gender	*Men*			-0.56	0.024		
	Women			Ref.			
**Private life**							
Household child(ren)	*Yes*	-0.24	0.019	-0.33	0.042		
	No	Ref.		Ref.			
Access to healthcare	*Dissatisfied/very dissatisfied*	0.45	0.001	0.48	0.017		
	Neither satisfied nor dissatisfied	0.14	0.282	0.46	0.019		
	Very satisfied/satisfied	Ref.		Ref.			
**Professional life**							
General factors							
School sector	*Public*			Ref.			
	Private			1.20	0.029		
School urbanicity	*Urban (A-B)*	Ref.					
	Intermediate (C-D)	-0.06	0.636				
	Rural (E-F)	0.36	0.004				
Physical environment							
Material conditions	*Unsatisfied (D-F)*	0.32	0.002				
	Satisfied (A-C)	Ref.					
Psychosocial environment							
Feeling of safety at school	*Rarely/not at all*	0.59	0.001	0.94	0.002	0.98	0.002
	Most of the time	0.01	0.917	0.43	0.036	-0.02	0.935
	Always	Ref.		Ref.		Ref.	
Victim of violence	*Yes*	0.30	0.005				
	No	Ref.					
Quality of relationships with students	*Low*	0.51	<0.001	0.54	0.006		
	Medium	0.16	0.270	-0.32	0.125		
	Strong	Ref.		Ref.			
Quality of relationships with colleagues	*Low*	0.18	0.134			0.24	0.368
	Medium	-0.35	0.007			-0.83	0.012
	Strong	Ref.				Ref.	
Quality of relationships with superiors	*Low*	1.77	0.004	0.68	0.003	0.91	0.003
	Medium	1.27	0.235	0.63	0.003	0.64	0.059
	Strong	Ref.		Ref.		Ref.	
**Organization / career perspectives**							
Autonomy at work	*Little/zero*	0.58	0.001				
	Some	0.03	0.797				
	A lot	Ref.					
Teamwork	*Rarely-never (D-F)*			0.53	0.003		
	Always-often (A-C)			Ref.			
Communication	*Rarely-never (D-F)*	0.27	0.028				

		France		Québec		Belgium	
% Poor work/life balance#		*14.6%*		*8.0%*		*9.2%*	
		**β**	**p-value**	**β**	**p-value**	**β**	**p-value**
	Always-often (A-C)	Ref.					
Salary	*Unsatisfied (D-F)*	0.34	0.048	0.50	0.004	0.52	0.013
	Satisfied (A-C)	Ref.		Ref.		Ref.	
Continuing education	*Unsatisfied (D-F)*	0.33	0.022				
	Satisfied (A-C)	Ref.					
Career progression	*Unsatisfied (D-F)*	0.76	0.017				
	Satisfied (A-C)	Ref.					

Weighted statistics taking into account gender and level of education + age group in France and Belgium

*Covariates that were not selected by the forward stepwise logistic regression procedure in any of the 3 country models of poor work/life balance: Household partner, Access to training/prevention resource, Age of students, Seniority, Employment status, Union membership, Remote work / School size, School facilities, Indoor air quality, Indoor noise level / Witness of violence / Societal appreciation

Table S5. Factors* associated with poor subjective health among French, Québec, and Belgian teachers, 2021 International Barometer of Education Personnel's Health and Wellbeing (I-BEST)

		France		Québec		Belgium	
% Poor subjective health#		*17.5%*		*7.2%*		*14.4%*	
		**β**	**p-value**	**β**	**p-value**	**β**	**p-value**
**Sociodemographic**							
**Age**		0.03	<0.001			0.03	0.003
**Private life**							
Household partner	*Yes*					-0.44	0.019
	No					Ref.	
Access to healthcare	*Dissatisfied/very dissatisfied*	0.42	0.001	0.78	<0.001	1.22	0.001
	Neither satisfied nor dissatisfied	0.28	0.015	0.77	<0.001	0.49	0.020
	Very satisfied/satisfied	Ref.					
Access to training/prevention resources	*No*	0.36	0.019				
	Yes	Ref.					
**Professional life**							
General factors							
Age of students	*3-5*					0.02	0.937
	6-11					-0.56	0.023
	11-15					0.29	0.173
	16-18 years old					Ref.	
Employment status	*Full-time*	Ref.		Ref.			
	Part-time	0.39	0.009	0.99	<0.001		
Union membership	*Yes*	Ref.					
	No	-0.02	0.820				
	Do not want to answer	0.49	0.006				
School sector	*Public*	Ref.					
	Private	0.39	0.035				
School size	*< 100*			Ref.			
	100-199			-0.63	0.100		
	200-799			-0.88	0.007		
	> 800			-0.85	0.015		
Physical environment							
Material conditions	*Unsatisfied (D-F)*	0.19	0.054				
	Satisfied (A-C)	Ref.					
Indoor noise level	*Unsatisfied (D-F)*	0.20	0.047			0.48	0.011
	Satisfied (A-C)	Ref.				Ref.	
Psychosocial environment							
Feeling of safety at school	*Rarely/not at all*	0.71	<0.001	1.24	<0.001	0.95	0.001
	Most of the time	0.25	0.037	0.56	0.013	0.44	0.070
	Always	Ref.		Ref.		Ref.	
Victim of violence	*Yes*	0.29	0.003			0.40	0.029
	No	Ref.				ref.	
Quality of relationships with students	*Low*	0.62	<0.001				
	Medium	0.30	0.032				
	Strong	Ref.					
Quality of relationships with colleagues	*Low*	0.45	<0.001	0.71	0.001	0.94	<0.001
	Medium	-0.15	0.233	0.28	0.187	0.14	0.625

% Poor subjective health#		France<br>*17.5%*		Québec<br>*7.2%*		Belgium<br>*14.4%*	
		**β**	**p-value**	**β**	**p-value**	**β**	**p-value**
	Strong	Ref.		Ref.			
Quality of relationships with superiors	*Low*	0.42	0.006	0.65	0.004		
	Medium	0.09	0.563	0.39	0.069		
	Strong	Ref.		Ref.			
**Organization / career perspectives**							
Autonomy at work	*Little/zero*	0.39	0.019	0.72	0.014	0.80	0.003
	Some	0.01	0.951	0.42	0.051	0.38	0.096
	A lot	Ref.		Ref.		Ref.	
Career progression	*Unsatisfied (D-F)*			0.48	0.009		
	Satisfied (A-C)			Ref.			

Weighted statistics taking into account gender and level of education + age group in France and Belgium

*Covariates that were not selected by the forward stepwise logistic regression procedure in any of the 3 country models of poor health: Gender, Household child(ren), Seniority, Remote work / School urbanicity, School facilities, Indoor air quality / Witness of violence / Teamwork, Communication, Societal appreciation, Salary, Continuing education

Table S6. Factors* associated with poor mental health among French, Québec, and Belgian teachers, 2021 International Barometer of Education Personnel's Health and Wellbeing (I-BEST)

		France		Québec		Belgium	
% Poor mental health#		*51.4%*		*35.3%*		*45.5%*	
		β	p-value	β	p-value	β	p-value
**Sociodemographic**							
Age		-0.01	0.013	-0.02	<0.001		
Gender	*Men*	-0.19	0.033	-0.29	0.031		
	Women	Ref.		Ref.			
**Private life**							
Household partner	*Yes*					-0.39	0.006
	No					Ref.	
Household child(ren)	*Yes*	-0.23	0.002	-0.22	0.022		
	No	Ref.		Ref.			
Access to healthcare	*Dissatisfied/very dissatisfied*	0.32	0.004	0.33	0.010		
	Neither satisfied nor dissatisfied	0.01	0.934	0.48	<0.001		
	Very satisfied/satisfied	Ref.		Ref.			
Access to training/prevention resources	*No*	0.49	<0.001			0.44	0.025
	Yes	Ref.				Ref.	
**Professional life**							
General factors							
Employment status	*Full-time*			Ref.			
	Part-time			0.30	0.032		
School sector	*Public*			Ref.			
	Private			0.93	0.021		
Psychosocial environment							
Feeling of safety at school	*Rarely/not at all*	0.91	<0.001	1.09	<0.001	1.23	<0.001
	Most of the time	0.17	0.032	0.35	0.001	0.47	0.001
	Always	Ref.		Ref.		Ref.	
Victim of violence	*Yes*	0.37	<0.001			0.41	0.005
	No	Ref.				ref.	
Witness of violence	*Yes*			0.25	0.009		
	No			Ref.			
Quality of relationships with students	*Low*	0.61	<0.001	0.59	<0.001	0.35	0.040
	Medium	0.14	0.137	0.27	0.014	-0.00	0.999
	Strong	Ref.		Ref.		Ref.	
Quality of relationships with colleagues	*Low*	0.25	0.009			0.47	0.005
	Medium	-0.05	0.583			0.10	0.554
	Strong	Ref.		Ref.		Ref.	
Quality of relationships with superiors	*Low*	0.24	0.027	0.70	<0.001	0.44	0.006
	Medium	0.15	0.151	0.36	0.001	0.34	0.039
	Strong	Ref.		Ref.		Ref.	
**Organization / career perspectives**							
Autonomy at work	*Little/zero*	0.58	<0.001			0.67	0.002
	Some	0.21	0.007			0.19	0.201
	A lot	Ref.				Ref.	

		France		Québec		Belgium	
% Poor mental health#		*51.4%*		*35.3%*		*45.5%*	
		**β**	**p-value**	**β**	**p-value**	**β**	**p-value**
Teamwork	*Rarely-never (D-F)*	0.20	0.014				
	Always-often (A-C)	Ref.					
Communication	*Rarely-never (D-F)*	0.18	0.031				
	Always-often (A-C)	Ref.					
Societal appreciation	*Disagree/strongly disagree*			0.37	0.047		
	Agree/strongly agree			Ref.			
Continuing education	*Unsatisfied (D-F)*	0.32	<0.001				
	Satisfied (A-C)	Ref.					
Career progression	*Unsatisfied (D-F)*			0.31	0.001	0.30	0.042
	Satisfied (A-C)			Ref.		Ref.	

Weighted statistics taking into account gender and level of education + age group in France and Belgium

*Covariates that were not selected by the forward stepwise logistic regression procedure in any of the 3 country models of poor mental health: Age of students, Seniority, Union membership, Remote work / School size, School urbanicity, School facilities, Material conditions, Indoor air quality, Indoor noise level / Salary

Table S7. Factors* associated with life satisfaction among French, Québec, and Belgian teachers, 2021 International Barometer of Education Personnel's Health and Wellbeing (I-BEST)

		France		Québec		Belgium	
% Life satisfaction#		*22.1%*		*42.6%*		*29.2%*	
		**β**	**p-value**	**β**	**p-value**	**β**	**p-value**
**Sociodemographic**							
**Age**						-0.02	0.010
Gender	*Men*			0.33	0.009		
	Women			Ref.			
**Private life**							
Household partner	*Yes*	0.29	0.011	0.37	0.001		
	No	Ref.		ref.			
Household child(ren)	*Yes*	0.22	0.023				
	No	Ref.					
Access to healthcare	*Dissatisfied/very dissatisfied*	-0.72	<0.001	-0.44	0.001	0.72	0.058
	Neither satisfied nor dissatisfied	-0.63	<0.001	-0.72	<0.001	-0.88	<0.001
	Very satisfied/satisfied	Ref.		Ref.		Ref.	
Access to training/prevention resources	*No*	-0.34	0.003	-0.18	0.050	-0.55	0.006
	Yes	Ref.		Ref.		Ref.	
**Professional life**							
General factors							
Employment status	*Full-time*	Ref.					
	Part-time	0.29	0.038				
Remote work	*At least partially*					0.35	0.032
	No					Ref.	
Physical environment							
Material conditions	*Unsatisfied (D-F)*	-0.30	0.001				
	Satisfied (A-C)	Ref.					
Indoor noise level	*Unsatisfied (D-F)*			-0.21	0.038		
	Satisfied (A-C)			Ref.			
Psychosocial environment							
Feeling of safety at school	*Rarely/not at all*	-1.19	<0.001	-0.37	0.141	-1.32	<0.001
	Most of the time	-0.29	0.002	-0.33	0.001	-0.63	<0.001
	Always	Ref.		Ref.		Ref.	
Victim of violence	*Yes*	-0.31	0.004			-0.37	0.046
	No	Ref.				Ref.	
Quality of relationships with students	*Low*	-0.74	<0.001	-0.86	<0.001	-0.76	<0.001
	Medium	-0.31	0.005	-0.32	0.002	-0.33	0.054
	Strong	Ref.		Ref.		Ref.	
Quality of relationships with colleagues	*Low*	-0.22	0.054				
	Medium	0.09	0.404				
	Strong	Ref.					
Quality of relationships with superiors	*Low*	-0.43	<0.001	-0.45	0.001	-0.73	<0.001
	Medium	-0.16	0.167	-0.25	0.031	-0.33	0.060
	Strong	Ref.		Ref.		Ref.	
**Organization / career perspectives**							

		France		Québec		Belgium	
% Life satisfaction#		*22.1%*		*42.6%*		*29.2%*	
		**β**	**p-value**	**β**	**p-value**	**β**	**p-value**
Autonomy at work	*Little/zero*	-0.87	<0.001	-0.89	<0.001	-1.25	<0.001
	Some	-0.54	<0.001	-0.10	0.304	-0.43	0.002
	A lot	Ref.		Ref.		Ref.	
Salary	*Unsatisfied (D-F)*	-0.77	<0.001	-0.41	<0.001	-0.43	0.002
	Satisfied (A-C)	Ref.		Ref.		Ref.	
Career progression	*Unsatisfied (D-F)*			-0.43	<0.001		
	Satisfied (A-C)			Ref.			

Weighted statistics taking into account gender and level of education + age group in France and Belgium

*Covariates that were not selected by the forward stepwise logistic regression procedure in any of the 3 country models of life satisfaction: Age of students, Seniority, Union membership, School sector / School size, School urbanicity, School facilities, Indoor air quality / Witness of violence / Teamwork, Communication, Societal appreciation, Continuing education
